# Supplementary material for: Patterns of Intron Gain and Loss in Fungi
Source: PLoS Biol. 2004 Nov 30;2(12):e422. doi: 10.1371/journal.pbio.0020422 (PMC532390; doi:10.1371/journal.pbio.0020422)
Supplement: Table S1 — Also available at http://genes.mit.edu/NielsenEtAl/. (4.3 MB ZIP). [file pbio.0020422.st001.zip › NielsenEtAl/html/1359.html]

AN3059.1.NCU02252.1.MG00901.1.FG06055.1


```
 CLUSTAL W (1.82) Multiple Sequence Alignments - Introns Inserted


Sequence 1: NCU02252.1	519 aa
Sequence 2: MG00901.1	512 aa
Sequence 3: FG06055.1	525 aa
Sequence 4: AN3059.1	520 aa
Alignment Length: 529 aa
Number Identitical Residues: 324 aa
Alignment Score (without introns) 15045


MG00901.1 	--------MDF1VIDGWGVASEESPKNGDAIAAAETPVMDEFA----RSKTGYVELEASS
NCU02252.1	-MAPEHKACLI1VIDGWGIPSEESPKNGDAIAAAETPVMDELS----KSATGFSELEASS
FG06055.1 	MSKTDQKACLI1VIDGWGIPSADSPKDGDAITNAKTPVMDALY----KDSKGYTELEASS
AN3059.1  	MAKVDQKVVLI1VIDGWGVAGPNSPKEGDAIAAAETPFMSGFAEENSKTAQGYTELDASS
          	    . .   : ******:.. :***:****: *:**.*. : ...::   *: **:***

MG00901.1 	LAVGLPEGLMGNSEVGHLNIGAGRVVWQDVVRIDQSIKTGEFSKNEVITQVIEAAKSGNG
NCU02252.1	LAVGLPEGLMGNSEVGHLNIGAGRVVWQDVVRIDQTIKKGELSQNEVIKATFERAKNGNG
FG06055.1 	LAVGLPEGLMGNSEVGHLNIGAGRVVWQDVVRIDQTIKNGELSQNDVIKKTFQSAAGGNG
AN3059.1  	LAVGLPEGLMGNSEVGHLNIGAGRVVWQDSVRIDQTLKKGELNKVENIAASFKRAKDGNG
          	***************************** *****::*.**:.: : *   :: * .***

MG00901.1 	RLHLCGLVSHGGV0HSKQEHLYSLLKAAKEAQVPKVFIHFFGDGRDTDPKSGVGYMEELL
NCU02252.1	RLHLCGLVSHGGV0HSKQTHLYALLKAAKEAGVPKVFIHFFGDGRDTDPKSGAGYMQELL
FG06055.1 	RLHLCGLVSHGGV0HAKQTHLYALLKAAKEYGVPKVIIHFFGDGRDTDPKSGAGYMQELV
AN3059.1  	RLHLLGLVSDGGV~HSNITHLIGLLKVAKEMEIPQVFIHFFGDGRDTDPKSAAKYMQQLL
          	**** ****.*** *::  ** .***.***  :*:*:**************.. **::*:

MG00901.1 	EKTKEIGIGQIATVVGRYYAMDRDKRWERVELAMKGLVLGEGEASEDPVKTVKERYEKG-
NCU02252.1	DTIKEIGIGELATVVGRYYAMDRDKRWERVEVALKGMILGEGEESTDPVKTIKERYEKG-
FG06055.1 	DNIKEIGVGEIGTVVGRYFAMDRDKRWDRLEIALKGLILGEGEASEDPVATVKARYEQGG
AN3059.1  	DATKEIGVGEIATVVGRYYAMDRDKRWDRVEIAIKGILSGEGEESSDPVKAIEKRYTED-
          	:  ****:*::.******:********:*:*:*:**:: **** * *** ::: ** :. 

MG00901.1 	--ENDEFLKPIVVGGDEGRIKD1GDNVFFFNYRSDRVRQITQLLGDVDRSPRPDFPYPKI
NCU02252.1	--ENDEFLKPIVVGGDERRIKE~DDTVFFFNYRSDRVRQITQLMGGVDRSPLPDFPFPNI
FG06055.1 	DMNKDEFLTPIIVGGDDRRIKD1DDTVFFFNYRSDRVRQITQLLGDVDRSPLPDFQYPKI
AN3059.1  	--ETDEFLKPIIFGGKDRRVQD~DDTLFFFNYRSDRVREITQLLGDYDRSPKPDLPYPKN
          	  :.****.**:.**.: *::: .*.:***********:****:*. **** **: :*: 

MG00901.1 	K-LATMTRYKLDYPFDVAFEPQKMGNVLAEWLGKQNVPQVHVAETEKYAHVTFFFNGGVE
NCU02252.1	K-LVTMTQYKLDYPFDVAFKPQQMDNVLAEWLGKQGVKQVHIAETEKYAHVTFFFNGGVE
FG06055.1 	KPLVTMTQYKVDYPFEIAFKPQHMGNVLAEWLGKQNVEQVHIAETEKYAHVTFFFNGGVE
AN3059.1  	IHITTMTQYKTDYTFPVAFPPQHMGNVLAEWLGKKDVQQCHVAETEKYAHVTFFFNGGIE
          	  :.***:** **.* :** **:*.*********:.* * *:****************:*

MG00901.1 	KAFALEERDESQDLVPSNKSVATYDKAPEMSADGVADQVAKRLGEQKFPFVMNNFAPPDM
NCU02252.1	KVFPLETRDESQDLVPSNKSVATYDKAPEMSADGVANQVVKRLGEQEFPFVMNNFAPPDM
FG06055.1 	KVFPLETRDQSQDLVPSNKSVATYDLAPEMSADGVADQVVKRLGEQKFPFVMNNFAPPDM
AN3059.1  	KQFPGEVR----DMIPSPK-VATYDLEPEMSAEGVGKKVEERIAEGKFEFVMNNFAPPDM
          	* *. * *    *::** * *****  *****:**..:* :*:.* :* ***********

MG00901.1 	VGHTGVYEAAIVGCAATDKAIGKIYEACKAHNYVLFITADHG~NAEEMKFPDGKPKTSHT
NCU02252.1	VGHTGVYEAAIVGCAATDKAIGKILEGCKKEGYILFITSDHG~NAEEMKFPDGKPKTSHT
FG06055.1 	VGHTGVYDAAVIGCEATDKAIGKILEGCKKAGYVLFITADHG~NAEEMKFADGKPKTSHT
AN3059.1  	VGHTGVYEAAIKGVAATDKAIGIIYEACKKHGYTLFITADHG2NAEEMLNEKGTPKTSHT
          	*******:**: *  ******* * *.**  .* ****:*** *****   .*.******

MG00901.1 	TNKVPFIMANAPEGWSLKPV-GGVLGDVAPTVLACMGLPQPEEMTGENLLVKA
NCU02252.1	TNKVPFIMANAPEGWSLKKE-GGVLGDVAPTILAAMGLPQPAEMTGQNLLVKA
FG06055.1 	TNKVPLLMANYPEGWSLKKTTEGVLGDVAPTVLAAMGLPQPEEMTGKSLLEKA
AN3059.1  	TNRVPFIMANAPEGWSLRKQ-PGVLGDVAPTVLAAMGIEQPKEMSGESLLVKA
          	**:**::*** ******:    *********:**.**: ** **:*:.** **
```
